# First-line Rucaparib Plus Bevacizumab Maintenance Completed One-Year in Germline BRCA1-Mutated Advanced Ovarian Cancer

**DOI:** 10.7759/cureus.32493

**Published:** 2022-12-13

**Authors:** Somnath Roy, Joydeep Ghosh, Sandip Ganguly, Bivas Biswas, Jaydip Bhaumik

**Affiliations:** 1 Department of Medical Oncology, Tata Medical Center, Kolkata, IND; 2 Department of Gynaecological Oncology, Tata Medical Center, Kolkata, IND

**Keywords:** advanced ovarian cancer, brca1 mutation, maintenance therapy, bevacizumab, rucaparib

## Abstract

The present case study showed the novel approach of Rucaparib and Bevacizumab as first-line maintenance therapy in germline BRCA 1 mutated advanced high-grade serous carcinoma of the ovary. A 56-year-old female with high-grade serous carcinoma of the ovary (ECOG PS1) was treated with carboplatin and paclitaxel in combination with Bevacizumab (CPB), followed by interval debulking surgery. Since the patient was germline BRCA 1 positive, after completion of adjuvant chemotherapy, she was kept on Rucaparib along with Bevacizumab. The patient achieved a complete response and has been leading a disease-free life for the past one year with maintenance therapy of Rucaparib + Bevacizumab, though the patient did experience a few adverse events, including one episode of grade 3 anaemia, occasional grade 3 asthenia, and grade 2 diarrhoea (CTCAE V-4) which was managed by gradual dose reduction of Rucaparib from 600 mg twice daily dose to 300mg twice daily dose. With dose alteration of rucaparib along with bevacizumab as maintenance, the patient continues to tolerate rucaparib and stay relapse-free from disease.

## Introduction

High-grade serous ovarian carcinoma (HGSOC) is the most common type of ovarian cancer and accounts for approximately 70% of total epithelial origin of ovarian malignancy. According to the recent GLOBOCAN 2020 data, India has the second highest number of ovarian cancer incidences (14.6%) after China (17.6%) [1]. Epithelial origins are associated with a poor prognosis due to their advanced stage at diagnosis and are difficult to treat. Less than 20% of patients with FIGO-classified Stage IV disease have an expected five-year overall survival. Moreover, high-risk patients have a median overall survival of fewer than two years [2].

Historically, the standard care of treatment for advanced, FIGO-classified Stage IIIc or IV, epithelial ovarian cancer includes primary debulking surgery (PDS) with the operative goal of achieving an optimal cytoreduction with minimal residual disease (<1 cm), followed by adjuvant platinum and taxane-based regimen [3,4]. However, all patients are not suitable for PDS, and survival advantage is only observed in cases where optimal cytoreduction is possible. Neoadjuvant chemotherapy (NACT) has emerged as an alternative for patients in whom PDS cannot be performed and/ or to decrease the rate of perioperative complications [5,6].

In patients with advanced ovarian cancer, bevacizumab, an anti-vascular endothelial growth factor (VEGF) antibody, in combination with carboplatin and paclitaxel (CPB) in treatment and with bevacizumab continued as maintenance therapy improved progression-free survival (PFS) compared to carboplatin and paclitaxel (CP) alone [7,8]. In 2018, US FDA (Food and Drug Administration) approved rucaparib, a poly(ADP-ribose) polymerase (PARP) inhibitor, for the maintenance treatment of recurrent epithelial ovarian cancer in patients who are in complete or partial response to platinum-based chemotherapy. This approval was based on the ARIEL3 trial and taking the mutational status into account, this trial included BRCA mutant ovarian cancer patients [9]. Looking into the benefit in improving PFS of both the drugs separately and also looking into the benefit of adding another PARP inhibitor with bevacizumab as a maintenance therapy in homologous recombinant deficiency (HRD) positive ovarian cancer patients, this present case shows the treatment outcome of this novel combination of rucaparib and bevacizumab as first-line maintenance therapy in a 56-year-old lady with germline BRCA-positive HGSOC.

## Case presentation

**Figure 1:**
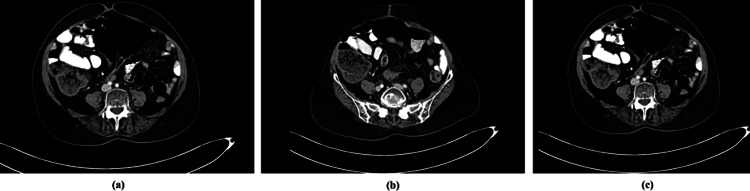
Contrast CT scan showed heterogeneous bilateral adnexal masses consistent with malignant neoplasm, omental and peritoneal metastatic deposits, ascites and enlarged retroperitoneal and pelvic nodes (brown arrow).

A 56-year-old female with ECOG PS1 was presented with progressive abdominal distension, pain and bloating sensation with decreased appetite since October 2020. A subsequent contrast-enhanced computerised tomography (CECT) scan revealed a right adnexal solid cystic mass, gross asities with multiple omental deposits along with left-sided cardio-phrenic nodes (Figures 1a-1c). During the laboratory examination, the CA 125 level was 2,685 U/mL and subsequent immunohistochemistry analysis revealed that the tumour cells were positive for WT-1 and PAX-8. The biopsy done for the omental deposit revealed FIGO-defined Stage IVB, HGSOC.

**Figure 2:**
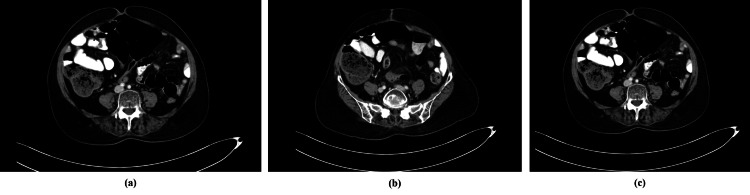
Contrast CT scan showed no pelvic mass or peritoneal nodules and no ascites.

She was immediately started on NACT with injection paclitaxel 175mg/m^2^ IV plus injection carboplatin AUC 6 IV every three weekly along with injection bevacizumab 7.5mg/kg IV every three weekly. In March 2021, post three cycles of NACT, she underwent interval debulking surgery. The post-operative histopathology report showed HGSOC, ypT3a N0 (AJCC 8th edition); all surgical margins were negative and there were no post-operative residual diseases (Figures 2a-2c). On subsequent analysis, germline BRCA1 mutation status turned out to be positive (pathogenic variant was detected in Exon 10 of BRCA1 gene; Strand Triesta Center for Cancer Genomics, Bangalore, India). In May 2021, she completed three more cycles of adjuvant paclitaxel carboplatin along with bevacizumab and then received maintenance bevacizumab at a similar dose of 7.5 mg/kg every three weekly for a total duration of 15 months. As she was germline BRCA1 gene mutation positive, since July 2021, tablet rucaparib 600 mg orally twice daily was added along with maintenance bevacizumab. During her treatment with maintenance rucaparib and bevacizumab, she experienced various adverse events, including one episode of grade 3 anaemia, occasional grade 3 fatigue, grade 2 diarrhoea along with grade 1 treatment-related emergent adverse events (CTCAE-V4) for which dose interruption for 21 days and gradual dose reduction to 500 mg, then to 400 mg and finally to 300 mg per orally twice daily was implemented. The patient achieved a complete response, and she was clinico-radiologically disease-free since the last follow-up in September 2022 and was taking rucaparib 300 mg twice daily without any interruption, completing one year of maintenance rucaparib.

## Discussion

Ovarian cancer is ranked as the seventh most commonly diagnosed cancer among women in the world and tumour biomarker plays a crucial role in diagnosis and determining the efficacy of the therapeutic treatment. A literature survey suggested tumour markers such as CA19.9 and CA 125 play crucial roles in the diagnosis of gynaecological tumours [10,11]. In the present case, the CA 125 level has been used to diagnose the stage of HGSOC.

Poly(ADP-ribose) polymerase (PARP) inhibitors have emerged to be a promising approach in the treatment of advanced ovarian cancer. Rucaparib is a small molecule PARP inhibitor that showed potent activity against PARP-1, PARP-2 and PARP-3. It has been approved by US FDA for treatment and maintenance therapy in adult patients with platinum-sensitive recurrent ovarian cancer [9,12]. In the randomised, placebo-controlled, phase III ARIEL3 trial, rucaparib at starting dose of 600 mg twice daily has significantly improved PFS when used as maintenance treatment. Median PFS in patients with a BRCA-mutant carcinoma was 16.6 months in the rucaparib group as compared to 5.4 months in the placebo group. Most common grade 3 adverse events include anaemia and increased alanine or aspartate aminotransferase concentration [9]. Further, the data from the ATHENA-MONO trial revealed that rucaparib monotherapy effectively acts as first-line maintenance therapy, providing significant clinical benefits in patients with advanced ovarian cancer with and without HRD [13].

Similar to rucaparib, another PARP inhibitor olaparib has shown significant efficacy as maintenance therapy in treating newly diagnosed advanced ovarian cancer with BRCA1/2 mutation [14] as well as in patients with platinum-sensitive relapsed ovarian carcinomas [15] In the phase 3 PAOLA-1 (PAOLA-1/ENGOT-ov25) trial, maintenance therapy with olaparib and bevacizumab was evaluated in patients with newly diagnosed HRD positive (including those without BRCA mutation) advanced ovarian carcinoma after platinum-based chemotherapy along with bevacizumab. The trial showed that the addition of maintenance olaparib to bevacizumab provided a significant PFS benefit mostly among HRD positive population [16]. Further, in the European Society for Medical Oncology (ESMO) Congress 2022, the final results from the phase III PAOLA-1 showed improved overall survival with the combination of olaparib and bevacizumab [17]. Likewise, the phase I MITO 25 trial was conducted to determine the pharmacokinetics, maximum tolerated dose and safety profile of rucaparib co-administered with bevacizumab as maintenance therapy for patients with high-grade ovarian cancer. The trial identified that the maximum tolerated dose of rucaparib is 500 mg twice daily when co-administered with bevacizumab. In addition, the combination did not reveal any new safety concerns [18]. The above literature review paves a new pathway towards the use of this novel combination of maintenance rucaparib along bevacizumab in patients with newly diagnosed HGSOC after first-line chemotherapy. Similar to this trial, in this present case, a 56-year-old female with a HGSOC was kept on a combination of bevacizumab and rucaparib as maintenance since the mutational status of the patient was germline BRCA1 positive. Initially, rucaparib was initiated at a dose of 600 mg twice daily, which was gradually reduced to 300 mg orally twice daily to minimize adverse events and improve tolerance. The combination of bevacizumab 15 mg/kg d1 q21d plus rucaparib 500 mg BID will be used in the multicentre phase II portion of MITO 25 trial in patients with newly diagnosed, high-grade epithelial ovarian, fallopian tube, or primary peritoneal cancer.

## Conclusions

Rucaparib has proven to improve survival outcomes in advanced ovarian cancer patients but also comes with a range of adverse events that need to be managed either by dose alteration and/or supportive care. In this case of germline BRCA1 mutated advanced HGSOC, the patient on maintenance therapy with the combination of rucaparib and bevacizumab showed a complete response and continues to tolerate a lower dose of rucaparib.
